# Between Online and Offline Solidarity: Lessons Learned From the Coronavirus Outbreak in Italy

**DOI:** 10.1177/00027642221132177

**Published:** 2022-11-01

**Authors:** Maria Laura Ruiu, Massimo Ragnedda

**Affiliations:** 1 Department of Social Sciences, University of Northumbria, Newcastle upon Tyne, UK; 2 Department of Arts, University of Northumbria, Newcastle upon Tyne, UK

**Keywords:** COVID-19, social solidarity, #andràtuttobene, #iostoacasa, Durkheim

## Abstract

This paper focuses on four e-initiatives that were precipitated by the coronavirus outbreak in Italy. These experiences played a relevant role in developing multilevel solidarity (from the local to the global level) both online and offline. They are represented by the hashtags “#iorestoacasa” (I stay at home) and “#andràtuttobene” (everything will be alright), “performances on the balcony,” “influencers’ campaigns,” and “altruism and e-parochialism.” These experiences represent revealing examples essential to understand the benefits that a mediated form of solidarity can produce. This is particularly important given the challenges that solidarity faces due to the technological acceleration imposed by the pandemic, which is likely to influence social relationships even in the post-pandemic era. Four lessons can be learned from these expressions of e-solidarity related to the capacity of Information and Communication Technologies to (1) promote unconditioned altruism; (2) fight “parochialism” when the same disadvantaged condition is shared; (3) their capacity to develop a multilevel sense of community by connecting the local experience to the global dimension; and (4) to mediate between institutional sources and people, and connect family members, friends, vulnerable people with neighbors, and the global community. This last point suggests that the pandemic has offered fertile ground for both mechanical and organic forms of solidarity to emerge. On the one hand, it created a collective conscience based on shared vulnerabilities and interdependence. On the other hand, it is based on individualization and diversity. Indeed, these examples of Durkheimian collective effervescence show the paradox of a form of collective individualized and mediated solidarity, which is typical of contemporary society.

## Introduction

This article uses social media phenomena during Italy’s 2020 COVID-19 lockdown to explore certain propositions about social solidarity and to ask how the encounter between a population facing a common threat across all social groups and statuses might reconcile Durkheim’s classic distinction between mechanical and organic solidarity. In other words, what happens when a highly differentiated society, bound organically, comes to universally share the same challenge conducive to a form of solidarity that Durkheim might describe as mechanical? This paper examines some experiences that can help understand this apparent paradoxical overlapping between mechanical and organic solidarity. Such forms of collective solidarity are mediated by the “technological individualisation” imposed by the pandemic, which is likely to influence social relationships even in the post-pandemic era. This is important to consider since social solidarity represents a powerful response to mitigate shock during crises (Mishra & Rath, 2020). Crises can cause psychological trauma due to a loss of attachment to places and their social function, which are core elements for the formation of collective identities (Erikson, 1976). However, the technological acceleration produced by the COVID-19 pandemic might have transformed social distancing in (an online) collective effervescence (Durkheim, 1915). Following Thijssen’s (2012) revision of the Durkheimian concepts of solidarity, this paper will interpret these forms of solidarity as an integrative process that links structural forces of integration with intersubjective sources of integration.

More specifically, this paper reflects on some forms of e-solidarity which have spontaneously emerged as a reaction to the COVID-19 outbreak by exploring a specific context represented by Italy. The Italian case represents a model for other countries on how to manage the social anxiety and isolation caused by lockdown. This is also evident in the emulation of the same initiatives in other European countries, such as Spain, France, and England, where lockdown arrived later. Italy was the first European country with a high number of COVID-19 cases compared to mainland China (WHO, 2020). Since the identification of the first two cases on January 31 2020, the Italian government has increasingly adopted restrictive measures to contain the spread of the virus. These measures included quarantine for over 50,000 people in eleven towns of northern Italy (Gazzetta Ufficiale, 2020a) on February 22 2020, and the lockdown of the entire country on the 10th of March (Gazzetta Ufficiale, 2020b).

Given the extreme decision to lock down the entire country, this paper investigates the effects produced by the restrictions in terms of the rise of e-solidarity initiatives. E-solidarity is intended as an (online) expression of a “particular solidarity” (Schwartz, 2007) that arises from specific circumstances and generates a collective emotional understanding. In this sense, solidarity is accomplished when individual emotions are made collective and individuals share empathy for each other (Stewart & Schultze, 2019). Such a group, created by social media participants, shares emotions related to the COVID-19 lockdown and signals a certain response or sensibility to it. More specifically, the shared emotion in this specific case is represented by benevolence, which underpins intergroup empathy (Louis et al., 2019). We studied patterns of online signaling that have been explicitly identified in Italy’s mass media and by international audiences as socially salient, and conducive to collective support. In turn, we studied the emergence and trajectory of those signals, by referring to the most popular collective forms of online reciprocal support that emerged during the first lockdown.

The fact that people were forced to stay at home under curfew for the very first time since the end of the Second World War, increased use of social media to look for information, communicate, and for entertainment. It also became fertile ground for the increase of e-solidarity initiatives that connected people at the micro-meso-macro levels, and also in the offline realm. At the family/friends’ level (micro level), social media allowed people to virtually meet up and either have aperitifs/coffee/dinner together or to take part in activities together (such as collective music videos). At the neighborhood level (meso level), social media was used to organize collective meetings and activities such as singing from the balconies. At the national level (macro level), some influencers launched initiatives and hashtags that connected people and encouraged them to respect the rules imposed by the central government.

To investigate these multilevel forms of solidarity, the first section reviews the literature related to e-solidarity. The second section reports on the research strategy adopted to identify the most popular e-solidarity initiatives. The third section reports the results of the analysis of four initiatives. These results will be discussed in relation to the role played by social media in supporting the organization and implementation of such collective activities. Finally, some conclusions will be drawn by highlighting that these forms of mediated solidarity exemplify the paradox of collective individualized solidarity, which might reconcile the coexistence of forms of both mechanical and organic solidarity.

## Literature Review

Solidarity can be defined as “the imperative to act towards vulnerable others without the anticipation of reciprocation” (Chouliaraki, 2011). While this definition requires the presence of both vulnerable subjects who receive solidarity and “distant others” who provide support, Fenton (2008, p. 49) refers to “a spirit of mutuality and reciprocity without individual advantage [. . .], leading to a network of individuals [. . .] that are bound to a political project.” This definition also seems to include people who share the same conditions and join efforts in a common political/social project. However, both definitions rely upon the individual choice of being part of a collective instance for the common good (Kolers, 2012). This interpretation of solidarity seems to challenge the structural interpretation of social solidarity as interpreted by Durkheim by emphasizing an interplay between individual self-reflexivity and structural determinants. However, the structural conditions imposed by the circumstances and the regulatory framework to tackle the pandemic are the basis for reconciling this apparent discrepancy. In fact, for Durkheim ([1893] 1969) mechanical solidarity is typical of pre-modern societies characterized by high degrees of cohesion. The division of labor and the complex organization of modern society led instead to an organic form of solidarity based on complementary differences. However, following Thijssen (2012, p. 456), “each of Durkheim’s structurally imposed forms of solidarity can ultimately be linked to subjectively based emotions and cognitions.” To reconcile this contradiction, Thijssen refers to intersubjective recognition, emphasized by Fraser and Honneth (2003), and the dialectic interpretation of solidarity proposed by Honneth (1996), who interprets intersubjective logic (antithesis) in connection to structural logic (thesis), which result in a synthesis of both an instinctual and rational identification with the structural principles of the group. However, this process does not necessarily have to be read as being in dialectical opposition. In this direction, the persistence of forms of mechanical solidarity in an organic society might be interpreted as a result of this interplay between intersubjective recognition and structural normalisation of solidarity and between more mechanical and spontaneous forms of reciprocity and instrumental aggregation of complementary forces in times of crisis.

The use of digital technologies during the pandemic might help understand this interplay thanks to their capacity to expand communication networks which are characterized by different durability, internal structure, and strength of ties (Stalder, 2013). On the one hand, they offer a platform for like-minded people to interact and potentially create a cohesive identity during crises when additional control is enforced. On the other hand, a recent review of studies (Waytz & Gray, 2018) suggests that online technology for communication may function both as a social connector and a separator. Online technologies have been found to promote impersonal communication (Konrath et al., 2011; White & Dorman, 2001) and individualism (Wellman et al., 2003). At the same time, online technologies functionally connect users who have different expertise and might connect instrumentally around a specific purpose. A line of studies shows that social media contributes towards building resilience in a time of crisis (Duffy, 2012; White, 2012; Yates & Paquette, 2011). Social media is generally recognized to facilitate both the flow of information between institutional sources and people (Vos & Sullivan, 2014) and connectedness with family/friends and to the broader community, by providing support and guidelines (Taylor et al., 2012). Moreover, it connects people in disadvantaged contexts to combat social isolation (Molyneaux et al., 2012).

García et al. (2010) identify different forms of cooperation, of which altruism (cooperation with everyone) and parochialism (cooperation with in-group members) might help understand the rise of integrated forms of mechanical and organic solidarity, but also collective and individualized expressions of solidarity during the Covid pandemic. However, among several factors, altruism results from direct benefits and reciprocal interactions that will have positive effects on the network (Lehmann & Keller, 2006). The concept of altruism is generally defined as an expression of benevolence (Louis et al., 2019) based on the welfare and happiness of others (Kelly & Walsh, 2015) or on the urge to help another (West et al., 2007). Altruism is defined by Durkheim as a characteristic of mechanical society, which is characterized by uniformity and sharing of ideas. Parochialism has recently received attention by scholars (Choi & Bowles, 2007; Lehmann & Feldman, 2008) who defined it as an individual sacrifice to benefit the in-group and harm an out-group. Therefore, parochialism is an expression of altruism that is in-group oriented and creates a collective conscience characterized by a unifying identity and internal cohesion. Even though the interpretation of organic solidarity has been sometimes contradictory in Durkheim’s work, especially when explaining the individualism that characterizes organic society (Bearman, 1991), altruism and parochialism might resolve this contradiction. In fact, especially when considering the emergence of a threat such as the pandemic, the function of such networks of interest might be temporarily instrumental. This highlights the individual interest in being part of an exchange (e.g., feeling of protection) against an external common threat. The empirical section of this paper will use some case studies to show how the first wave of COVID-19 triggered both these two forms of altruism (unconditioned and parochial) also thanks to the use of social media. Therefore, given these premises and the sometimes-conflicting debate around the forces that generate mechanisms of solidarity, there is still a need to investigate the role of the Internet, and social media in particular, in supporting the expression of (e)solidarity.

This aspect deserves attention to understand what virtuous mechanisms might be activated in a time of crisis in terms of solidarity networks, which, in turn, might be reproduced when the crisis is over. In this vein, this paper aims to answer the following research questions:

RQ1: *What are the positive outcomes activated by e-solidarity mechanisms in Italy during the first wave of the COVID-19 pandemic?*

Previous studies show that users tend to use social media to express their concerns and closeness to people affected by a crisis (Hjorth & Kim, 2011; Procopio & Procopio, 2007; Smith, 2010). However, as highlighted by this literature review, the development of “in-group solidarity” might mirror “out-group hate” (Yamagishi & Mifune, 2009) and parochialism (García et al., 2010). In fact, solidarity might also trigger the emergence of groups that act as independent agents in their own interest (Turchin, 2003). The in-group and out-group dynamics might suggest activation of mechanical solidarity thanks to the development of a collective conscience around a common threat that increases internal cohesion. Therefore, the second research question aiming at investigating how altruism can lead to the formation of in- and out-groups, and mechanical and organic mechanisms of solidarity, is articulated in two sub-questions:

RQ2a: has COVID-19 e-solidarity generated in-group versus out-group dynamics?
RQ2b: How can the COVID-19 multilevel (local-national-global) and individualized (through individual use of technologies) mechanisms of e-solidarity resolve the dichotomous distinction between mechanical and organic solidarity?

## Research Strategy

We studied patterns of online signaling which had been explicitly identified in Italy’s mass media and by international audiences as socially salient and conducive to collective support. Specifically, in the early stages of lockdown, we identified four main cases of e-solidarity that are represented by online networks of support that were amplified by mainstream media at the national and international levels: (i) the hashtags “#iorestoacasa” (I’m staying at home) and “#andràtuttobene” (everything will be all right) on social media, (ii) “performances on balconies,” (iii) “influencers’ campaigns,” and (iv) “altruism and e-parochialism.” The two hashtags were chosen because on the 10th of March 2020 the Government enforced the law decree #iorestoacasa (Ministero della Salute, 2020) and both hashtags were among the most used during the first lockdown in Italy and were associated with messages of hope and positive emotions (Otte, 2020). Between the 10th and the 24th of March 2020, the hashtag “iostoacasa” was used 1.4m times on Instagram, plus 264k uses of similar hashtags (such as, e.g., #restiamoacasa). In the 2 weeks considered, 554 videos were posted on YouTube with the hashtag #iorestoacasa and 472 videos with the hashtag “andràtuttobene,” 186 of which either involved children as protagonists or were targeted at children. On Instagram, the hashtag “andràtuttobene” appeared 582k times, and 627k similar hashtags (such as, e.g., “andratuttobene” or “tuttoandràbene”) were used.

The performances on the balcony were chosen because they have spread across the world following the Italian example to promote reciprocal encouragement (Fidler, 2020; Taylor, 2020). Ferragni’s celebrity campaign was chosen because it raised more than 4m euros that were used to build a new intensive care unit at the San Raffaele hospital (Burioni, 2020). Finally, we will discuss the main examples of e-parochialism that emerged during the first lockdown. This analysis was based on multiple sources including national reporting by the most read national newspaper (*La Repubblica*, Statista, 2019), YouTube videos, and Instagram posts (published between the 10th and 24th of March 2020) that contain the two hashtags. *La Repubblica* dedicated an online page to update news about the Covid-19 hour by hour (Stabile & Matteucci, 2020).

The analysis starts on the 10th of March 2020 (when the lockdown law decree came into force) and includes the observation of the first 2 weeks of lockdown in Italy. Two weeks was sufficient to observe the consolidation of some dynamics, especially considering that thanks to the Internet, other countries had started to adopt the same practices when their governments established lockdown. Finally, the study involved an interview with one of the “performers” to further investigate her commitment to solidarity practices.

All these cases represent per se expressions of solidarity and the aim of the present study is not to quantify and classify their different social effervescence. In contrast, this research adopts a qualitative case study methodology, using various data sources to explore the solidarity phenomenon within its naturally occurring context (Rashid et al., 2019). The case studies were selected to observe the specific dynamics that characterize each of these forms of reaction in a period of crisis and understand how these examples have become successful in alleviating social isolation across the world. In other words, these expressions of online collective effervescence unite people who are physically distant but share the same goals (Schiermer, 2014). These examples also help explain the coexistence of multilevel forms of solidarity that, as this paper will try to show, from a theoretical point of view might be interpreted as a reconciliation between mechanical and organic forms of solidarity.

## Case Studies

This section explores the main characteristics of the four cases included in this paper. It is split into four parts, following the four main forms of e-solidarity generated through social media, but amplified by mainstream media, both nationally and internationally.

### #iorestoacasa and #andràtuttobene

The hashtag #iorestoacasa was launched on Twitter by a member of the Democratic Party Filippo Sensi (@nomfup). It became so popular that the law decree, which entered into force on the 10th of March 2020 and locked down the entire country, was named after it. Moreover, it was translated into several languages (in French as #jerestealamaison, in Spanish as #quedateencasa and in English as #stayathome), when other European countries were forced to adopt the same restrictive measures (Bozza, 2020). Together with #andràtuttobene, it became an identifier for several indoor activities and experiences posted on social media. However, #iorestoacasa tended to be associated with both adults and children, and with many different activities such as cooking, reading, artistic performances, singing, fitness activities, and makeup demonstrations. Several videos were also posted by the National TV channel Mediaset (2020) and other channels such as Spettacolomania.it (2020), FanPage.it (2020), and Jose Antonio Naranjo Mena de Andújar (2020) in which celebrities promoted auto-isolation.

In contrast, #andràtuttobene was more likely to be associated with children’s messages and activities. In fact, some mothers in the South of Italy posted pictures and videos on Facebook of pictures painted by their children using rainbow colors and the message “everything will be alright.” The hashtag became viral across the country. However, the use of this hashtag originated offline before the lockdown was put in place. In fact, a poet launched an initiative to leave post-its around Milan with the message “everything will be alright” (Landoni, 2020). Then, this practice spread across the country and was advertized on social media. Several initiatives originated from this message. In fact, the “Zecchino d’oro” festival, the most popular children’s singing competition in Italy, promoted a song titled “Everything will be alright (Let’s stay together),” and invited children to download the material and learn and sing the song online. The hashtag became so popular that, on the 12th of March 2020 a headline in *The Guardian* translated the message of the tag #andràtuttobene in “Everything will be alright” and classified it as a “message of hope” (Otte, 2020). Moreover, after the UK was put under lockdown, on the BBC (2020b) a schoolteacher encouraged her alumni and other children to draw a rainbow, which is always associated with the hashtag. Some videos also showed international solidarity such as those posted by the Embassy of Romania (Ambasciata di Romania nella Repubblica Italiana, 2020) in which some Embassy operators applauded Italy (and its workers on the frontline). In a video realized by the International Group for Technical Cooperation with Developing Countries (Il Dolomiti, 2020), Ugandan Children sang “Come on Italy,” and in a video posted by the Sustinente Council (Comune di Sustinente, 2020) children from Bangladesh wished Italy a swift recovery.

The use of hashtags might be considered both a mechanical and organic signal of solidarity. In fact, on the one hand they create individualized participation in the collective effervescence thanks to technologies, by creating a space for mechanical mechanisms of solidarity. This might be supported by the emergence of social cohesion against a common threat and in support of the restrictions. On the other hand, they create a platform for different expertise to meet and ensure the organic function of society based on the exchange of complementary competencies. In the above-mentioned examples, users played a role in activating mechanisms of mechanical solidarity thanks to connections with similar people who share the same worries. At the same time, the use of hashtags triggered complex organic forms of solidarity given the expertise offered to the members of that network. For example, teachers who instruct children to carry out some activities by labeling their expert competence with the hashtag #andràtuttobene might be interpreted as an organic form of solidarity, which supports both children who are home schooling and alleviates parents’ home-caring activities. On the other hand, children’s online participation is fundamental for education to function, especially when schools were closed (from the 10th of March to the 3rd of May 2020). Moreover, the various activities promoted online offered multiple expressions of expertise at the service of the collective.

### The Balcony Performances

The balcony performances included several activities such as music and collective singing, individual performances (professional musicians and dancers) and fitness activities. However, the most popular activity was collective singing. *La Repubblica* titled an article “Italy on the balcony: songs against fear” (Scorza, 2020). This initiative, which was spontaneously called “flash-mob” on social media, was launched on Facebook by Stefania Cammarata di Mango, a singing teacher from Turin, on the 13th of March 2020. From that day, every afternoon at noon people on their balconies/rooftops/windows across the country met to applaud the Italian doctors, nurses, and workers serving on the frontline, and every evening at 18:00 to sing songs together (Tortello, 2020). These arrangements became so popular that *The Guardian* labeled the initiative as “balcony singing in solidarity” (Thorpe, 2020); the BBC (2020a) reports that “Italy sings to defy coronavirus,” *The New York Times* defined it as “a Moment of Joy” in a “Moment of Anxiety” (Horowitz, 2020), El Pais (2020) defined it as a reaction of the Italian spirit and *Le Figaro* as a way to replace suspended cultural events (AFP, 2020). For example, in a singing performance, a woman in Turin involved her older neighbor, who could not meet in person, repeatedly shouting “Ciao Vittoria, I love you” (Marta Frida, 2020). The performer was virtually interviewed about her relationship with her neighbor. She said that she and her partner started to do the shopping for their older neighbor and to have a daily relationship with her through social media. In exchange, the neighbor supported them with her expertise in cooking traditional food from the South of Italy (where her family is originally from), strengthening their relationship.

This activity is also connected to some celebrity campaigns aimed at promoting respect for the restrictions. In fact, several singers and artists performed on their balconies or in their homes. In this case too, the boundary between mechanical and organic solidarity becomes thin. The balcony singing became a sort of ritual, which might be interpreted as a form of mechanical solidarity that generates “group identification and identity fusion” (Whitehouse & Lanman, 2014). On the other hand, it is also an organic form of solidarity that keeps basic functions of society at work. This is exemplified by the substitution of cultural events with balcony performances by celebrities, which keep cultural and artistic functions of society active in exchange for an audience. At the same time, this form of solidarity provided the opportunity for individual creativity to emerge and functional use of the media to activate social solidarity, oriented to keep society functioning.

### Influencers’ Campaigns

Among the most effective social influencers’ campaigns, the “Go Fund Me” campaign launched by Chiara Ferragni (an Instagram influencer) and her husband Fedez (a rapper) in collaboration with Professor Alberto Zangrillo (head of the cardiovascular and intensive care department at Milan’s San Raffaele hospital) was particularly successful (https://www.gofundme.com/f/coronavirus-terapia-intensiva). In 4 days, the campaign raised more than 4m euros used to build a new intensive care unit at the San Raffaele hospital (Burioni, 2020).

On the 19th of March, another campaign was launched by the same couple in collaboration with the singer Andrea Boccelli to collect 100k euros to buy equipment for a hospital in Camerino (Il Fatto Quotidiano, 2020). Moreover, other celebrities posted on Instagram or shared their songs written as a sign of solidarity for the tragedy that hit Italy. For example, U2 vocalist Bono (@u2) shared the song “Let Your Love Be Known” written for Italy’s coronavirus victims (Chilton, 2020). Another successful example by the group Casa Surace was shared across Facebook, Twitter, Instagram, and YouTube and, at that time, amassed more than 3m followers (Casa Surace, 2020). This family and friends-based experiment started in 2015 as a way to post funny videoclips that mock the rivalry between the North and the South of the country. Their video, in which the grandmother gives advice on how to prevent the spread of the virus, was so popular that it was subtitled in several languages (e.g., English, Arabic, Farsi, and Chinese), shared by Aljazeera (2020), Daily Mail (2020), Breakfast Television Toronto (2020), and mentioned by UK newspapers (Giuffrida, 2020).

Even though these examples might depoliticise and reduce solidarity practice to individualized acts of technological consumption, by also producing an uncertain impact on individual moral consciousness in the long run (Driessens et al., 2012), in the case of COVID-19, everyone was affected and was aware of what was happening. Therefore, even interpreting the fundraising campaign promoted by Ferragni and Fedez as a form of “marketization of philanthropy” (Nickel & Eikenberry, 2009) or as a form of “charitainment” (Dayan & Katz, 1992), this action raised the money needed to construct an intensive care unit, which was functional to the management of the pandemic. In this sense, this mechanism of solidarity might be interpreted as typical of an organic society in which the entertainment function of celebrities activates collective efforts to adapt to concrete need for resources. At the same time, it activates mechanical solidarity through social media by creating the conditions for users to share the sentiment of belonging to a country that is under pressure (Anderson, 2020).

### Altruism and E-parochialism

Some forms of racism were perpetrated in Italy against Chinese people before the virus spread in the country (The Guardian, 2020). Furthermore, when Italy was still the only country in Europe to be affected by the outbreak, some Internet users from other countries made light of the Italian situation, for example, by sharing a video published by a private French television channel (Canal +) about “corona pizza.” Even though this video was immediately removed, the “parochial altruism” was amplified by Italian responses, with the publication of several videos on YouTube. However, when the virus spread to France and other countries, people started to emulate Italy’s example by, for example, performing on the balcony to express their solidarity to both in-group and out-group people (Fidler, 2020). Therefore, following Durkheim ([1893] 1969), we may argue that sharing the same problem might be interpreted as a trigger for passing from a mechanical form of solidarity (within a small group that shares similar conditions) to a form of organic solidarity (exchange and interest-driven interdependencies within society). Moreover, this transition might also be explained by the interactive character of digital technologies (Ragnedda & Ruiu, 2020). Indeed, the fact that users can share and interact in real-time favors dialogue and reciprocal support when the emergency involves everyone. However, this type of reciprocal support is also functional to the management of the crisis and creates interdependencies between those who have accumulated expertise and those who need an antecedent to learn how to manage the crisis. Moreover, in this context, social media is not used to preserve local cultures, as shown by previous research (Molyneaux et al., 2012), but rather local culture is used to strengthen a sense of belonging to the country. This multilevel sense of community (from the neighborhood to the national level) also serves as a testimony of resilience that connects other cultures and countries in similar conditions. For example, social media also enabled people from China and Italy to connect and support each other. This is also evidenced by several exchanges of supportive messages on *YouTube* between the two countries, such as in the case of Italian people who thank Chinese people for their help from their balconies (New China TV, 2020a), or post individual videos (New China TV, 2020b), and Chinese people supporting Italy to “stay strong” (New China TV, 2020c).

## Discussion

The four examples of solidarity, all triggered by the coronavirus restrictions, can be classified as examples of digital collective “swarms.” Such swarms are collective and spontaneous actions implemented by independent individuals in pursuit of a collective goal (Stalder, 2013). This suggests that individuals fought against their isolation by virtually participating in a collective effort to support each other. In response to RQ1, the outbreak generated e-solidarity mechanisms that simultaneously work on two levels, which suggest the coexistence of both forms of mechanical and organic solidarity in the COVID-19 pandemic. One level relates to the in-group, which includes people connected through physical proximity or friendship/blood ties (e.g., people living in the same neighborhood who meet up every day on their balconies, or families). This example might be interpreted as an expression of mechanical solidarity dictated by a common enemy (the pandemic) and shared social norms that condemn actions that are not accepted by the collective conscience (Fraser & Honneth, 2003) and might put the collective at risk. A second level relates to the out-group (e.g., people with no personal connections but who share the same problem, and people who are altruist but not directly affected by the problem). In this direction, previous studies show that users tend to express their concerns and closeness to people affected by a crisis on social media (Hjorth & Kim, 2011; Procopio & Procopio, 2007; Smith, 2010). In the present case, people were simultaneously motivated by an individual need to collectively alleviate the negatives deriving from forced isolation, and by a form of altruism towards those who were more isolated than others (e.g., vulnerable people) and could not meet other people for any reason. For example, the above-mentioned singing performance in Turin might be interpreted as a form of organic solidarity in which instrumental solidarity emerges by connecting people with different functions and different backgrounds. This informal network of support played a fundamental role during the pandemic in supporting the formal Italian social support system, which has been subject to budgetary constraints (Sanfelici, 2020). At the same time, instrumental does not mean unemotional. In fact, it requires intersubjective relationships that represent a fertile ground for mutual empathy (Thijssen, 2012).

Differently from previous findings (Bernhard et al., 2006; Wit & Wilke, 1992; Yamagishi et al., 1999), which showed how community members tend to collaborate with in-group members more than with “outsiders,” the Italian lockdown shows that e-solidarity can work simultaneously towards both “parochial altruism” (Choi & Bowles, 2007; Lehmann & Feldman, 2008) and “unconditioned” altruism (Lehmann & Keller, 2006; Nowak, 2006). In the first case, Internet technologies served as an appropriate tool for developing neighborly dynamics, combating social isolation and organizing practical activities (social meet-ups and activity programs). In the second case, digital technologies are used to connect and share that specific experience (e.g., singing from balconies) with outsiders (through the publication of videos on social media platforms), and in turn, invite other people and communities to do the same.

However, in response to RQ2, this latter form of e-solidarity is triggered by sharing the same disadvantage, which reinforces the emergence of mechanical forms of solidarity based on in-group cohesion. The e-parochialism between countries which were already hit by the virus and those which were not, reinforces the idea that mechanical solidarity can be found in conjunction with organic solidarity because it is instrumental to the management of the problem. In fact, when other countries were also affected by the virus, this parochialism tended to disappear because of a rationally and emotionally motivated exchange of knowledge and experience. Moreover, this multilevel expression of e-solidarity was reinforced by “celebrity humanitarianism” (Chouliaraki, 2011) that efficiently used the Internet to perform, and emotionally express their own “humanity” by substituting the voice of the “suffering other.” The “celebrity humanitarianism” simultaneously enhanced in-group dynamics by fundraising for hospitals and vulnerable people, and out-group mechanisms by creating awareness about the problem globally. The example of Casa Surace is effective in this direction. The use of well-known expressions from Neapolitan dialect and cultural stereotypes serves to reinforce the connections between the local culture (in-group) and the national identity (out-group). Moreover, its translation into other languages serves as a bridge between different cultures and societies that have the same problem. Finally, among the tips provided by the video, the grandmother also suggests that young people should teach the elderly how to use social media to take care of them virtually.

Therefore, even though the cooperative capacity of a group might result from different attitudes and individual behaviors (García et al., 2010), altruism and e-solidarity in the case of coronavirus result from reciprocal interactions that benefit the network (Lehmann & Keller, 2006). Social media promoted and facilitated communication processes not only between institutional sources and people (Vos & Sullivan, 2014), but also between family members and friends and with the broader community (Taylor et al., 2012). Social media also offered the opportunity for vulnerable people to alleviate social isolation (Molyneaux et al., 2012). Anderson (2020) interpreted the panic caused by the pandemic as a trigger for mechanical solidarity because the individual is connected to the collective consciousness and collective ideas/behavior (also due to a more repressive system of control). We argue that the use of online technologies generates a paradox of individualized collective solidarity, which in turn is a synthesis of both mechanical and organic tendencies. These examples reinforce the “individualized” character of contemporary forms of collective solidarity, which increasingly appear to be anonymous, mediated, and decentralized (Schiermer, 2014). This paradox suggests that both forms of solidarity (mechanical and organic) are not mutually exclusive. The persistence of mechanical solidarity in organic society suggests that structural and individual determinants are at play in this dialectic. The structural constraints (the pandemic and its related “repressive rules”), led to the emergence of solidarity that is simultaneously intersubjective, instrumental, emphatic, instinctive, collective, and individualized. The decision to engage in mediated practices of particularistic forms of solidarity (identification with group identity) and universalistic solidarity (instrumental intersubjectivity) via the Internet suggests that technologies become a fertile ground for the integration of mechanical and organic forms of solidarity. Despite the individualistic tendency that characterizes the use of technologies, the case studies show that they can humanise solidarity, but they can also be functional to the system and goal oriented. This is also supported by the fact that, even when further waves have caused additional lockdowns in specific regions in Italy, the collective effervescence of balconies and the use of hashtags and social media live events (also celebrities’ live) progressively disappeared (Angelini, 2020; Culicchia, 2020). The “normalisation” of the crisis has softened the mechanical character of solidarity by showing its organic function, which was instrumental in managing the “panic” of the crisis.

## Conclusion

Some lessons can be learned from the four e-initiatives arising from the coronavirus outbreak in Italy and worldwide. First, Information and Communication Technologies represent a useful tool for fighting isolation and promoting unconditioned altruism. The virus outbreak triggered e-solidarity mechanisms at both in-group and out-group levels. The real-time interactive character of digital technologies favors dialogue and reciprocal support when the emergency involves everyone. This is directly connected to the second point related to a potential rise of “parochial altruism” between “affected” and “not-affected” groups. The coronavirus case showed that sharing the same disadvantages are more likely to activate solidarity and the emulation of practices. In turn, this is connected to the third lesson learned from coronavirus solidarity. This relates to the possibility of developing a multilevel sense of community (from the neighborhood to the national and global level), promoted not only by the intervention of “celebrity humanitarianism,” but also by people’s interaction and virtual sharing of experiences, performances, and worries. Finally, in this multilevel interaction, social media serves as a powerful tool for mediating between institutional sources and people, and for connecting family members, friends, vulnerable people with neighbors, and the global community.

In-group and out-group dynamics suggest that mechanical solidarity has been produced thanks to a collective conscience around a common threat that increases internal cohesion and also depends on the enforcement of social distance restrictions. At the same time, the individual experience and decision to become a member of these virtual networks might depend on instrumental reasons based on complementary differences that make people dependent on each other. Exploring four examples of e-solidarity, we tried to show that solidarity can be interpreted dialectically through the synthesis of the structural aspect (thesis) and the intersubjective logic (antithesis). This synthesis incorporates both instinctual and rational identification with the structural principles of the group. Therefore, this instinctual and rational choice corresponds to the simultaneous activation of mechanical and organic solidarity, which in turn helps explain the paradox of individualized collective forms of solidarity. This means that the “technological individualisation” imposed by the pandemic, has transformed the crisis into an online collective effervescence, which was functional in maintaining society’s organic functions. This also suggests a link between structural (necessity) and intersubjective (agency) sources of solidarity that are powerful in times of crisis.
